# Midwinter Arctic leads form and dissipate low clouds

**DOI:** 10.1038/s41467-019-14074-5

**Published:** 2020-01-10

**Authors:** Xia Li, Steven K. Krueger, Courtenay Strong, Gerald G. Mace, Sally Benson

**Affiliations:** Department of Atmospheric Sciences, University of Utah, 135 S 1460 E, Salt Lake City, UT 84112-0102 USA

**Keywords:** Atmospheric dynamics, Attribution, Cryospheric science

## Abstract

Leads are a key feature of the Arctic ice pack during the winter owing to their substantial contribution to the surface energy balance. According to the present understanding, enhanced heat and moisture fluxes from high lead concentrations tend to produce more boundary layer clouds. However, described here in our composite analyses of diverse surface- and satellite-based observations, we find that abundant boundary layer clouds are associated with low lead flux periods, while fewer boundary layer clouds are observed for high lead flux periods. Motivated by these counterintuitive results, we conducted three-dimensional cloud-resolving simulations to investigate the underlying physics. We find that newly frozen leads with large sensible heat flux but low latent heat flux tend to dissipate low clouds. This finding indicates that the observed high lead fractions likely consist of mostly newly frozen leads that reduce any pre-existing low-level cloudiness, which in turn decreases downwelling infrared flux and accelerates the freezing of sea ice.

## Abstract (web summary)

Cracks in Arctic sea ice (leads) are becoming more prevalent and widespread, yet studies regarding their impacts on clouds are limited. Here, contrary to the present understanding, diverse observations and modelling simulations show that higher leads concentrations do not necessarily result in more low clouds.

## Introduction

Leads are quasi-linear openings within the interior of the polar ice pack, where the ocean is exposed directly to the atmosphere^1^. Leads range in width from several meters to tens of kilometers with narrow leads most abundant and in length from hundreds of meters to hundreds of kilometers (Supplementary Fig. 1). Due to the extreme air–water temperature contrast (20–40 °C), turbulent heat fluxes over leads can be two orders of magnitude larger than over the ice surface in winter^2^. While winter leads climatically cover 2–3% of the total surface area of the central Arctic and 6–9% of the Arctic peripheral seas (Supplementary Figs. 2 and 3), these large heat fluxes dominate the wintertime heat budget of the Arctic boundary layer^2–5^. Moreover, leads are also a source of moisture which can induce boundary layer clouds locally^6^. The presence of these clouds has the potential to extend the thermodynamic impact of leads over large-scale regions through their vertical and horizontal development^7,8^ as well as the enhanced downward infrared radiative flux^9–11^. Boundary layer cloud cover also exerts a significant impact on the equilibrium sea ice thickness^10^, which is a key indicator of changes in Arctic heat transport^4^. Therefore, low-level clouds significantly influence how leads impact the Arctic surface energy balance.

Correctly understanding the lead-modified surface energy budget is important. However, accurate determination of wintertime low-level cloud fraction over the Arctic Ocean from satellites is still a challenge owing to the presence of the underlying leads and sea ice, and the polar night. Previous observations demonstrate a replacement of thick, multi-year ice by thin, first-year ice^12^, a gradual acceleration in sea ice drift and wind stress^13^, increases in sea ice mean strain rate and the associated deformation rate^14^, and a marked widening of the marginal ice zone^15^ in the Arctic. All these observational findings suggest an increasing presence of Arctic leads in the climate system, which motivates a study to understand associated changes in boundary layer clouds and thus estimate the associated large-scale heat balance in response to changes in lead fraction. As a first step in this direction, here we explore the potential influences that leads have on boundary layer clouds in the wintertime Arctic.

Due to the limitation of observations in the Arctic, previous studies have mostly used model simulations to examine the clouds or convection induced by a single lead in idealized or simplified situations^8,11,16–23^. Few of those studies investigated the low-level clouds over a realistic ensemble of leads. In this study, diverse ground- and satellite-based observed records of the Arctic system near Barrow, Alaska, are combined with a three-dimensional cloud-resolving model to examine the effects of leads on boundary layer clouds. Based on the observations, we first attempt to establish a statistical relationship between the large-scale sensible heat flux due to leads and low-level cloud frequency. Furthermore, a cloud-resolving model is used to better understand the underlying physics of the observed lead-low cloud associations. We find low-level cloud occurrence frequency decreases with increasing large-scale lead flux and show that it is induced by the recently frozen leads, which constitute a majority of the satellite-detected lead fraction.

## Results

### Observed lead-low cloud associations

**Fig. 1:**
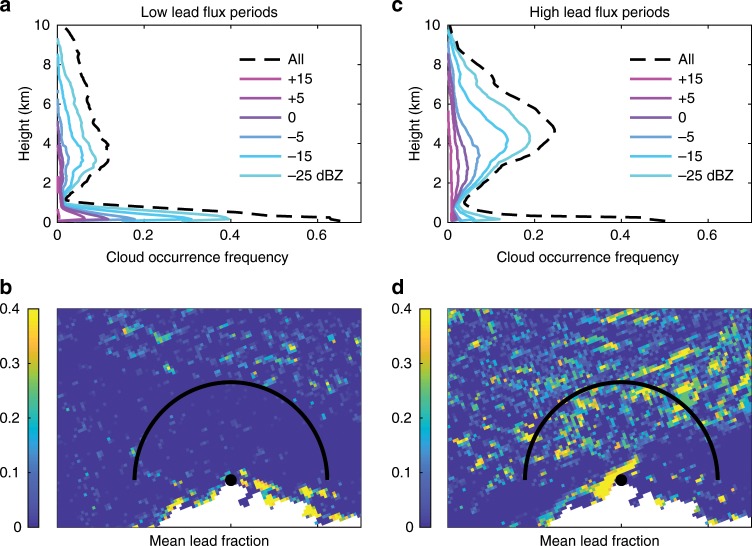
MMCR derived cloud occurrence frequency and the corresponding mean lead fraction. **a** The vertical frequency distribution of cloud occurrence as a function of minimum reflectivity (dBZ) for the low lead flux periods. **b** Mean lead fraction based on AMSR-E data for four representative low lead flux periods in February 2011 (days 14, 15, 26, 27). **c** The same as **a** but for the high lead flux periods. **d** The same as **b** but for four representative high lead flux periods in February 2010 (days 19, 22, 23, 24). The coastal region of Alaska is shown in white, Barrow is indicated by a filled black circle, and the top of the 200-km radius semicircle is directed north.

Previous observational analysis^24^ shows that low-level clouds predominate at Barrow throughout the year, especially during fall and winter. In this study, we investigate the effects of leads on low-level clouds at a local scale, offshore within 200 km of the Barrow site (as indicated by the black semicircle in Fig. 1b, d) over the period 2008–2011 (see Methods). Profiles of the MMCR (Millimeter Wavelength Cloud Radar) derived cloud occurrence as a function of reflectivity (dBZ) for the composites of low and high large-scale lead flux are shown in Fig. 1a, c, respectively. Based on prior research^25^, clouds are generally associated with dBZ < −10, while drizzle and light precipitation (heavy precipitation) correspond to dBZ between −10 and 10 (dBZ ≥ 10). Current understanding^16,20^ suggests we would find more boundary layer clouds under high lead flux conditions, but we find just the opposite in our analyses. The curves in Fig. 1c show that for the high lead flux intervals, clouds within the boundary layer (i.e., 0–1 km) with dBZ > −25 are observed to have a frequency of <10%. However, for the low lead flux periods, up to 40% of the boundary layer clouds have dBZ > −25 (Fig. 1a).

To investigate the robustness of this finding, we used the combined CloudSat-CALIPSO (Cloud-Aerosol Lidar and Infrared Pathfinder Satellite Observations)^26,27^ data to obtain profiles of cloud occurrence frequency in the region offshore within 200 km of Barrow and performed a similar analysis for the same composites of low and high lead flux intervals. The low-level cloud occurrence frequency (Supplementary Fig. 4) from CloudSat-CALIPSO over this region also decreases as the large-scale lead flux increases. The consistency of lead and low cloud associations between the MMCR and CloudSat-CALIPSO cloud occurrence frequencies suggests that the low-level clouds detected by ground-based remote sensing at Barrow are also representative of the low-level clouds over the adjacent ocean when the wind is onshore and of moderate speed (see Methods).

**Fig. 2:**
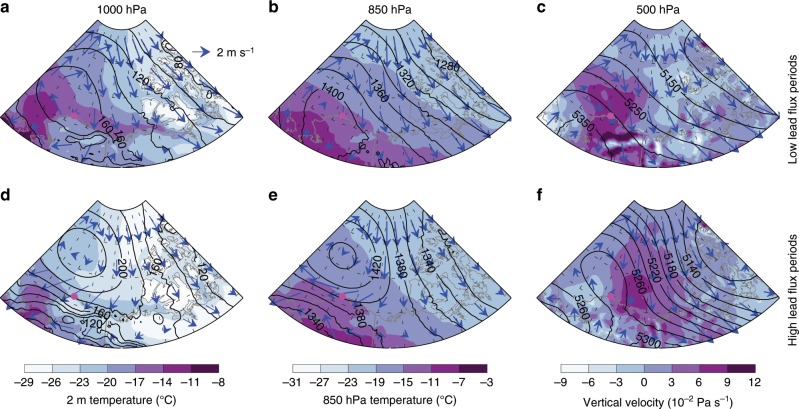
Large-scale synoptic conditions. **a**–**c** Composites of geopotential height (black contours) and wind velocity (arrows) at 1000 hPa (wind is at 10 m), 850 and 500 hPa for the set of low lead flux periods. Color shading indicates 2-m air temperature, 850-hPa temperature, and vertical velocity, respectively. **d**–**f** The same as **a**–**c** but for the high lead flux periods. The domain is 65–85 °N, 90–180 °W, and the Barrow site is indicated by the filled magenta circle.

The reliability of this unexpected result depends on effectively separating the effects of large-scale meteorology on the clouds and leads from the effects of leads on the low-level clouds. Therefore, we performed an analysis of the synoptic environments over a Barrow-centered region (Fig. 2) and pan-Arctic (Supplementary Fig. 5) for low and high lead flux periods, using hourly MERRA-2 (Modern-Era Retrospective analysis for Research and Applications version 2) reanalysis dataset^28^. The composite analyses for the low and high lead flux periods show similar patterns of geopotential height at each level (1000, 850, and 500 hPa). In both cases, a ridge tilts equatorward with height near Barrow and the adjacent ocean region, though it is slightly weaker in the composite of low lead flux intervals. The eastern flank of the ridge produces northerly wind for our study area. Overall, the two sets of periods have similar meteorological conditions, which minimizes the likelihood that the large-scale meteorology is significantly different between the two sets. The corresponding analysis using the NCEP-NCAR reanalysis produces results very similar to Fig. 2 (not shown). We also examined the meteorological conditions based on in-situ surface measurements at Barrow and the lead fractions that we used to calculate the large-scale lead fluxes (Supplementary Fig. 6). The 2-m air temperature, 10-m wind speed and wind direction have similar distributions in the high and low lead flux periods (Supplementary Fig. 6a–c, e–g). Among all the factors that contribute to the large-scale lead flux, it is found that lead fraction dominates the variations in large-scale lead flux, with a correlation of 0.9 (Supplementary Fig. 6d). This further demonstrates that meteorological conditions in both regimes resemble each other and cannot account for the differences between these two selected large-scale lead flux regimes.

### Investigating the underlying mechanisms

**Fig. 3:**
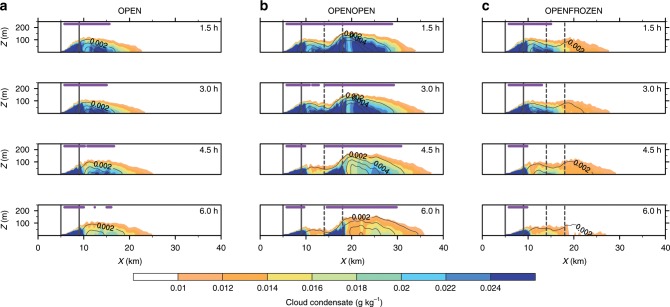
Evolution of the simulated clouds response to leads. **a** The *x*–*z* profiles of the cloud condensate (cloud water plus cloud ice mixing ratio, shaded contour lines) and precipitating water (rain and snow, solid contour lines with intervals of 0.002 g kg^−1^) from the OPEN case. **b**–**c** The same as **a** but for the OPENOPEN and OPENFROZEN cases, respectively. For each case, four simulation times (1.5, 3.0, 4.5, and 6.0 hrs) are displayed. The purple bars near the top of the selected domain indicate the place where the clouds are radiatively significant, with a total water path greater than 3.0 g m^−2^. All results are averaged along the *y* direction. The upstream open lead extends from *x* = 5 km to *x* = 9 km, as indicated by the two vertical solid lines in the domain, and the downstream open or frozen lead extends from *x* = 14 km to *x* = 19 km, as indicated by the two vertical dashed lines. Note that the scales are different on the *x* and *y* axis.

**Fig. 4:**
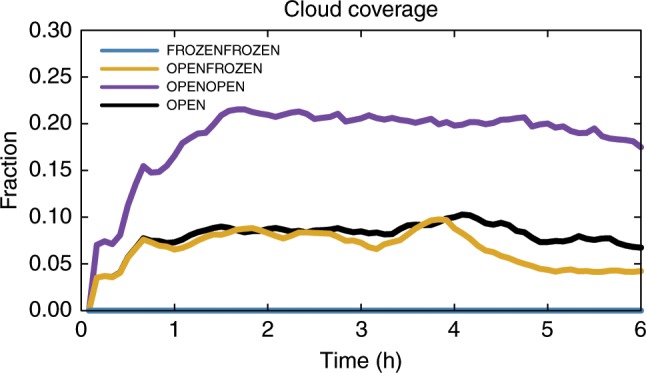
Time series of cloud coverage. Time series of the cloud coverage from the OPEN (black), OPENOPEN (purple), OPENFROZEN (orange), and FROZENFROZEN (blue) cases. The cloud coverage is defined as the fraction of grid points with radiatively significant clouds for the entire domain.

The surprising observational results suggest some intriguing physical mechanisms. For example, the dearth of low-level clouds in the high lead-flux case could reflect desiccation by precipitation or down-mixing of dry air by lead-induced convection. Three-dimensional cloud-resolving simulations (see Methods) were conducted in an effort to understand the underlying physics. The simulated boundary layer clouds from the four cases (i.e., OPEN, OPENOPEN, OPENFROZEN, and FROZENFROZEN, see Methods) are displayed in Figs. 3 and 4. The FROZENFROZEN case is not shown in Fig. 3 because no radiatively significant clouds are generated in this case, as shown in Fig. 4. The *x*–*z* hydrometeor profiles (Fig. 3) illustrate the cloud evolution in each case, while the time series of cloud coverage (Fig. 4) further quantitatively indicates the differences of cloud amount between these cases. Here a threshold of 3.0 g m^−2^ is chosen in Figs. 3 and 4 as downwelling LW radiation is particularly sensitive to total water path exceeding 3.0 g m^−2^, resulting in an increase in downwelling LW radiation by roughly 6 W m^−2^ compared to the initial condition. In both OPEN and OPENOPEN cases, extensive clouds are produced downstream of the lead (Fig. 3), which is consistent with previous studies^16,20,22^. A comparison of these two cases demonstrates that more boundary layer clouds are generated if the lead (completely ice-free) area fraction is doubled. The corresponding 6-h averaged cloud fraction is 0.185 in OPENOPEN versus 0.080 in OPEN (Fig. 4). This is what we expected based on current understanding. However, if the lead fraction is doubled but the added lead area is frozen (i.e., OPENFROZEN), lead-induced low clouds decrease to 0.066 (Fig. 4). At 6 h, cloud coverage in the OPENFROZEN case is 0.042, which is 37.3% less than that in the OPEN case. These differences of cloud fraction between the OPEN and OPENFROZEN cases are consistent with our observational analyses in which higher lead flux periods are associated with lower cloud occurrence frequencies, and also indicate that newly frozen leads tend to reduce the low-level clouds generated by an upstream open lead.

**Fig. 5:**
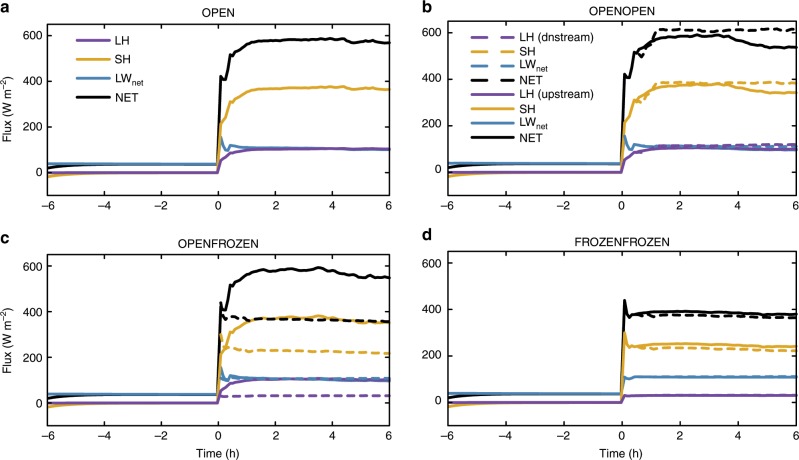
Time series of the surface fluxes averaged over lead area. **a** Time series of surface latent heat flux (LH), sensible heat flux (SH), net longwave radiative flux (LW_net_), and the net total flux (NET) averaged over the lead area from the OPEN case. NET excludes conductive heat flux at the top layer, and upward flux is defined as positive. **b** The same as **a** but for the OPENOPEN case. Solid curves indicate the fluxes averaged over the upstream open lead while dashed lines indicate the fluxes averaged over the downstream open lead. **c** The same as **b** but for the OPENFROZEN case with an upstream open lead (solid lines) and a downstream frozen lead (dashed lines). **d** The same as **b** but for the FROZENFROZEN case.

Because of the different thickness of ice between recently frozen leads and open leads (5 cm versus 0 cm in our simulations), the surface fluxes may change significantly. We analyzed the changes of surface fluxes to understand why a recently frozen lead can have such a significant impact on the evolution of clouds. To emphasize the impacts of leads on the surface energy budget, all the energy exchange components were averaged over the lead area (Fig. 5). As a comparison, over the thick ice surface (i.e., –6 to 0 h), we find longwave radiative fluxes dominate the surface heat exchange, with turbulent latent heat (LH) and sensible heat (SH) fluxes being close to zero. Over the open lead area, all the energy fluxes are significantly increased to different extents (Fig. 5a–c). Specifically, the 6-h averaged LH and SH in the OPEN case increase to 100 and 357 W m^−2^, respectively. The magnitudes of the counterparts over open leads in OPEN, OPENOPEN and OPENFROZEN cases are quite similar. However, these surface fluxes change in different ways over the frozen leads in OPENFROZEN and FROZENFROZEN. Comparing the turbulent fluxes over downstream frozen lead to upstream open lead in OPENFROZEN (Fig. 5c), we find that LH decreases sharply by 69% to 31 W m^−2^ due to the exponential dependence of the saturation vapor pressure upon temperature, while less reduction occurs in SH (decreased by roughly 35.8% to 228 W m^−2^). The differences in turbulent fluxes over open leads and frozen leads suggest that the turbulent fluxes (SH and LH) over the newly frozen leads play a key role in dissipating low-level clouds.

**Fig. 6:**
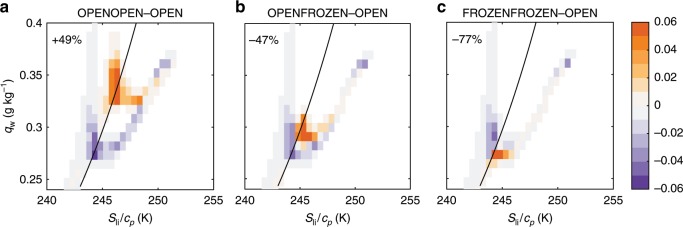
Mixing diagram of the difference between high and low lead fraction cases. **a** Differences of PDFs of thermodynamic state characterized by liquid-ice static energy (*S*_*li*_*∕c*_*p*_) and total water mixing ratio (*q*_*w*_, sum of the water vapor, cloud water, and cloud ice) between OPENOPEN and OPEN. Plotted results are from the last two simulation hours for all model grid volumes within a selected domain with *x* extending from 20 to 30 km and *z* extending from the bottom level to 300 m, where most clouds occur. The solid black line indicates the theoretical cloudy-clear boundary line, with cloud ice mixing ratio equal to 0.01  g kg^−1^. **b** The same as **a** but for differences between OPENFROZEN and OPEN case PDFs. **c** The same as **a** but for differences between FROZENFROZEN and OPEN case PDFs. In the OPEN case, *x* extends from 10 to 20 km. The percentages on the top left are the percentage changes of cloudy volume fraction from the OPEN case to the other three cases.

Changes of the 2D PDFs of the thermodynamic state characterized by liquid-ice static energy and total water mixing ratio from the OPEN case to the other three cases are displayed in Fig. 6. The corresponding PDFs for each case are shown in Supplementary Fig. 7. Results are from a 10 km by 12.8 km by 300 m domain with grid volumes of 200 m by 200 m by 12 m. We sampled the simulation results every 5 min, and calculated the PDF of each thermodynamic state bin for the last two hours. The PDF difference indicates an ~49% increase in the cloud volume fraction from the OPEN to OPENOPEN cases (Fig. 6a), owing to the shift of a large number of grid volumes to a larger total water mixing ratio and larger liquid-ice static energy from below to above the cloudy-clear boundary (black curve). This is caused by the large vertical turbulent temperature flux (Supplementary Fig. 8) and water vapor flux (Supplementary Fig. 10) over the two open leads in the OPENOPEN case. However, the fraction of cloudy grid volumes decreases by 47% from OPEN to OPENFROZEN. While a large number of grid volumes shift to a higher liquid-ice static energy, increases in total water mixing ratio are not sufficient to maintain all of the previously cloudy grid volumes (Fig. 6b). The reduced water vapor supply (Supplementary Figs. 10, 11) as well as the relatively large temperature flux (Supplementary Figs. 8, 9) over the frozen lead, which provides the necessary buoyancy for convection and entrainment of warm air, might play a central role in reducing the relative humidity and thus dissipating these low clouds. As for the difference between FROZENFROZEN and OPEN (Fig. 6c), almost all the previously cloudy grid volumes in the OPEN case are shifted downward and below the cloudy-clear border due to the reduced water vapor flux, which is consistent with the zero cloud coverage shown in Fig. 4. Our model simulation results provide a plausible explanation for the counterintuitive observational results: instead of including a larger fraction of completely open leads, the observed high lead fraction must largely consist of newly frozen leads which tend to reduce any pre-existing low-level cloudiness.

## Discussion

Our observational analyses indicate an unexpected lead-low cloud association and our numerical experiments show supporting results, that a region with an observed high lead fraction likely contains many newly frozen leads with ice thickness ranging from roughly 2.5 cm up to 30 cm, which largely suppress the latent heat flux with lesser impact on the sensible heat flux^29^ and consequently tend to dissipate boundary layer clouds (Supplementary Figs. 12 and 13). This explanation is reasonable given that open leads usually freeze over after remaining open for just a few hours and thinner ice generally grows faster than thicker ice. Consider a scenario in which no low-level clouds and no leads exist initially, after which leads open and produce low-level clouds. As new leads keep opening and then freezing, the recently frozen lead area will increase, which contributes to an increasing total detected lead area (e.g., Advanced Microwave Scanning Radiometer for EOS (AMSR-E) detected lead fraction). Because the accumulated newly frozen leads tend to dissipate low-level clouds, the large detected lead fraction is accompanied with fewer low clouds. As shown in Fig. 3, the cloudiness between an upstream open lead and a downstream open or frozen lead is decreased. Additionally, doubling the open lead fraction from OPEN to OPENOPEN does not necessarily double the cloud coverage (i.e., the increase is nonlinear, Fig. 4). These results indicate potentially important lead–lead interactions which will be further examined in our future work. We further compared the surface energy fluxes, averaged over the thick ice surface and within half of the entire domain (*x* = 0 to *x* = 51.2 km), from the OPENOPEN and OPENFROZEN cases, and find that with a lead fraction of 15.6%, freezing half the lead area and the consequent dissipation of low-level clouds could result in an increase in energy loss from the thick sea ice by ~1.6 W m^−2^. The entire domain averaged surface fluxes in each case (Supplementary Fig. 14) indicate the impacts of the different lead scenarios on the large-scale surface energy budget, compared to the NOLEAD case. These preliminary findings stress the importance of differentiating open water leads from recently frozen leads, though our simulated cloud coverage is less than that observed owing to the idealized configurations (e.g., invariable lead fraction, periodic boundary layer conditions, and neglect of moisture advection). Future work will pursue such differentiation and will also examine the effects of lead modulation of low-level clouds on the lead-atmosphere feedbacks and the large-scale surface heat balance over the pan-Arctic.

As mentioned above, besides the local surface-based process (i.e., lead fraction change), the large-scale meteorology may also have impacts on the low-level clouds, such as the temperature and humidity advection by the large-scale synoptic flow^30–33^. An analysis of 11 years of observations at NSA (North Slope of Alaska)^34^ showed that distinct synoptic-scale and mesoscale meteorology regimes produce distinct cloud states. For example, high pressure over the Beaufort Sea and the Arctic Ocean with northerly cold, dry anticyclonic flow causes fairly small cloud occurrence frequencies at all levels, while cyclonic flow around Aleutian low pressure systems leads to large cloud occurrence frequencies throughout the column by advecting warm, moist air through the Bering Strait. In our study, the Barrow radiosonde data and surface measurements were used to select two categories with similar atmospheric states, so that the influences of leads on the low-level clouds dominate. However, the reader should bear in mind that a role of the large-scale synoptic conditions in cloudiness cannot be excluded with great certainty, to which the slight difference between the cloud occurrence frequency in high levels above the boundary layer (Fig. 1a, c) may in part be related. Determining the relative roles of large-scale meteorology and large-scale lead fluxes in determining the distribution of low-level clouds will be addressed in our future studies. The modeling study demonstrates that recently frozen leads with a certain range of ice thickness tend to dissipate low clouds produced by open leads. It is hypothesized that both the suppressed water vapor supply and the strong convection induced by the large sensible heat flux provide favorable conditions for the dissipation of the clouds. Details of the processes that determine how low clouds are reduced by frozen leads, such as entrainment of warm air owing to the lead-induced convective plumes, will be further examined in future work.

## Methods

### Data and algorithm

In this study, we focus on an Arctic region offshore within 200 km of Barrow because of the relatively long-time record of Arctic clouds collected at Barrow, the NSA’s main research site, by the Atmospheric Radiation Measurement (ARM)^35^ program. The datasets used for the observational analyses are summarized in Supplementary Table 1. Observations for the months of January–April and November–December for the years 2008–2011 were chosen because of the availability of reliable data. For the present analyses, control for meteorological conditions, including large-scale flow regime, is a potential challenge which we address by using a conditional sampling algorithm (described below) as well as examining the large-scale synoptic pattern from reanalyses. Using the twelve-hourly radiosondes and hourly surface measurements^36^ at Barrow along with the daily lead fractions from the AMSR-E^37^, we first selected two subsets of the 12-h time intervals (centered at hour 6 or 18) that have similar atmospheric states at Barrow and over the adjacent ocean except for the large-scale turbulent sensible heat flux due to the leads. Then we compared the cloud occurrence frequency profiles for these two subsets using the NSA MMCR^38^ data at Barrow.

Specifically, we first calculated three quantities for all 12-h periods for which the needed data were available: dry fraction is the fraction of the atmosphere below 2000 m above ground level with relative humidity <50% at Barrow; turbulent surface flux from leads is the turbulent surface sensible heat flux per unit area over leads calculated from surface temperature of water (i.e., –2 °C), air temperature, surface pressure, relative humidity, and wind speed at Barrow following the previous work^39,40^; large-scale turbulent surface flux due to leads is the turbulent surface sensible heat flux from leads multiplied by the lead fraction (i.e., "large-scale turbulent sensible heat flux”). The lead fraction was derived from the AMSR-E lead area fraction for the Arctic dataset^37^ by averaging values within the semicircle north of Barrow (Fig. 1b). The AMSR-E lead detection method^37^ produces a lead area fraction which is the sum of open leads and those with thin ice cover by using the ratio of brightness temperatures observed at 89–19 GHz. Therefore, both open water leads and thin-ice-covered leads are included in the lead fraction.

All 12-h intervals were then conditionally sampled based on the following three criteria: a dry fraction >0.42 to avoid deep cloud layers that could make lead modulation of cloudiness difficult to detect, a wind direction between 240° and 110° (clockwise) to select trajectories that are from the ocean and over leads when present, and a wind speed >2.7 m s^−1^ (i.e., 117 km 12 h^−1^) to select trajectories that have crossed a substantial proportion of the lead-fraction-analysis region (which extends 200 km from Barrow) during the 12-h analysis period.

From the set of all available 12-h intervals that met these three criteria, two subsets were chosen: one with the component of large-scale turbulent sensible heat flux due to leads in the upper third of all such values ("high lead flux regime,” with large-scale flux >8 W m^−2^) and the other with this flux in the lower third of all such values ("low lead flux regime,” with large-scale flux <3.8 W m^−2^). A total of 91 samples (8.0%) were included for high lead flux intervals and 31 (2.6%) for the low lead flux intervals. Finally, the cloud occurrence frequency profiles were calculated for the two subsets using NSA MMCR data, processed with quality control and masking following the previous study^41^, to determine the statistical associations between large-scale lead flux and low-level cloud occurrence. The lead-low cloud associations were further examined using satellite data (offshore within 200 km of Barrow) from the CloudSat Cloud Profiling Radar (CPR)^26^ combined with CALIPSO lidar^27^. To characterize the large-scale synoptic environment for the two subsets, we used both the hourly MERRA-2^28^ and the six-hourly NCEP (National Centers for Environmental Prediction)–NCAR (National Center for Atmospheric Research) atmospheric reanalysis^42^.

### Model and experiment design

The model we used is the System for Atmospheric Modeling, version 6.11 (SAM)^43^, which is a three-dimensional, nonhydrostatic, cloud-resolving model. SAM employs appropriate physical parameterizations for subgrid-scale processes and is well-suited for simulating boundary layer clouds^44–46^. The infrared radiative fluxes interact with clouds and the surface as well as the atmosphere and provide radiative heating rates. The subgrid-scale turbulence closure employs a first-order Smagorinsky closure scheme in which the stability is accounted for in the levels above the surface layer; the surface turbulent fluxes are estimated using Monin-Obukhov similarity^47^ and more details about the integral forms of stability functions used in the Monin-Obukhov similarity are shown in the Supplementary Information. We used a two-moment ice-phase microphysics parameterization^48^. The horizontal domain size in our study is 102.4 km by 12.8 km with a horizontal grid spacing of 200 m. In addition, the model has 81 levels in the vertical direction with the model top at about 1.5 km, which is high enough to simulate the Arctic boundary layer. The vertical grid size is variable, with a minimum size of 12 m at the surface and an average size of 18 m. SAM uses periodic lateral boundaries, and a rigid lid at the top of the domain.

The Simplified Land Model (SLM)^49^ is coupled to SAM version 6.11 and is used to represent land-atmosphere interactions. SLM currently has 17 land surface types, including water and snow/ice. To simulate a recently frozen lead, we added a new land type, which is a 5-cm layer of ice covering open water. A land type index in each surface grid point automatically defines the parameters of the vegetation and soil. We set up a horizontally inhomogeneous surface with different land types to simulate the Arctic sea ice with leads. SLM also has an interactive soil model, currently with nine layers. We therefore used nine layers in the sea ice. For simplification, no snow is included in the present study. The vertical ice layer thickness is prescribed for each surface type, and the initial temperature profile is the equilibrium profile for the initial atmospheric conditions. In the SLM, the surface temperature of the ice evolves in response to the surface energy balance.

Because the AMSR-E lead area fraction includes both open leads and newly frozen leads, we designed a series of simulations to investigate the possible impacts of frozen leads on the boundary layer clouds. We began with a “NOLEAD" case (simulation) where the entire domain was covered by a single sea ice land type with a thickness of 1.995 m. This simulation was conducted and run for 6 h (–6 to 0 h) to ensure that the atmosphere and sea ice reached an equilibrium state. Then, corresponding to our observational analyses with different lead fractions, we performed simulations for four different lead configurations, where all leads are 4 km wide: an “OPEN" case in which a single lead was opened in the domain to investigate the effects caused by the presence of the open lead, an “OPENOPEN" case which modifies the OPEN case by adding another identical open lead 5 km downstream, an “OPENFROZEN" case which modifies the OPEN case by adding an identical but frozen lead 5 km downstream, and a “FROZENFROZEN" case which modifies the OPENFROZEN case by having both leads be frozen. The lead area fraction doubled from the OPEN case to the other three cases, but with different frozen lead proportion (i.e., 0%, 50%, and 100% in the OPENOPEN, OPENFROZEN, and FROZENFROZEN, respectively), which allows us to examine the potential impacts of frozen leads as well as lead fraction on the low clouds. All four cases were run for 6 h (0–6 h). Initial conditions were based on the Surface Heat Budget of the Arctic (SHEBA) project following previous work^8^, with the large-scale wind direction approximately perpendicular to the lead orientation. Here ensemble simulations with slightly different variation in initial conditions are not considered because the simulated flow is strongly forced by fluxes from leads instead of variability associated with initial conditions.

## Supplementary information

Supplementary Information

## Data Availability

The authors declare that the observational and reanalysis data supporting the findings of this study are available within the paper and its supplementary information file. Data from our model simulations are available upon request.

## Code availability

The codes used to generate these results are available upon request.
