# Supplementary material for: Midwinter Arctic leads form and dissipate low clouds
Source: Nat Commun. 2020 Jan 10;11:206. doi: 10.1038/s41467-019-14074-5 (PMC6954259; doi:10.1038/s41467-019-14074-5)
Supplement: Supplementary file 1 — Supplementary Information [file 41467_2019_14074_MOESM1_ESM.pdf]

Supplementary Information

"Midwinter Arctic Leads Form and Dissipate Low Clouds"

by Li et al.

Supplementary Methods 1

Method to calculate large-scale lead flux

"Large-scale lead flux" in the present study is defined as the contribution of leads to the entire domain sensible heat flux, and is calculated as the surface sensible heat flux over leads per unit area multiplied by the domain lead area fraction. The data used to calculate turbulent sensible heat flux over leads are from the in-situ surface measurements at Barrow. Because the conditional sampling algorithm (see Methods) ensures that trajectories within each 12-h analysis period are from the ocean and leads when present, and can crossed a substantial proportion of our domain region, it is reasonable to assume that the surface measurements at Barrow can represent those over the offshore ocean region. The method used to calculate the turbulent sensible heat flux over open water and thin ice covered leads are the same, and both follow the previous studies [1,2]. The method applies for the convective heat transfer over wintertime leads. Since the calculated sensible heat fluxes are weakly dependent on mean wind speed under the condition of free convection, roughness length is not used in the method. We also checked our results against previous work [2]. The main equation to calculate turbulent sensible heat flux is

$$H_{\mathrm{s}}(X) = \rho c_p D C_* \frac{\Delta T}{\Delta z_T} \tag{1}$$

where X is the lead width and is held constant at 3 km, ρ is the air density computed from the equation of state of ideal gases, c_p is the specific heat capacity of air, D is the molecular diffusivity of heat in the air, ΔT is the temperature difference between surface and reference altitude r ($r = 10$ m), C_* is the empirical non-dimensional coefficient, and is determined as a function of stability over the lead as:

$$C_* = \frac{0.3}{0.4 - h/L} + 0.15 \tag{2}$$

$$h = 0.82 \ln X + 0.02 \tag{3}$$

$$L^{-1} = 8.0(\frac{0.65}{r} + 0.079 - 0.0043r)Ri_B \tag{4}$$

$$Ri_B = -\frac{rg}{\overline{T}}\frac{\Delta T}{{U_r}^2} \tag{5}$$

Here h is the thermal internal boundary layer height over leads as a function of lead width, L is the Obukhov length, and is calculated using the method in previous study[3], Ri_B is the bulk Richardson number referenced to height r ($\overline{T}$ is the average of temperature between the surface and reference height).

Δz_T is the heat length scale that takes into account the kinematic viscosity of air (ν) and the buoyancy difference between the surface and the reference altitude (ΔB), and is defined by:

$$\Delta z_T = (\frac{\nu D}{\Delta B})^{1/3} \tag{6}$$

$$\Delta B = \frac{g}{\overline{T}}(\Delta T + \frac{0.61\overline{T}\Delta Q}{1 + 0.61\overline{Q}}) \tag{7}$$

Here ΔQ and $\overline{Q}$ are the difference and average of the specific humidity between surface and the reference height, respectively.

Supplementary Methods 2

Stability functions in Monin-Obukhov similarity in SAM

In the SAM model, the surface turbulent fluxes are estimated using Monin-Obukhov similarity. Following Monin-Obukhov similarity theory, integral forms of the stability functions for momentum (Ψ_{M}) and heat (Ψ_{H}) are

$$\Psi_{\mathrm{M}} = \begin{cases} 2\log\left(\frac{1+x}{1+x_0}\right) + \log\left(\frac{1+x^2}{1+x_0^2}\right) - 2\left(\tan^{-1}(x) - \tan^{-1}(x_0)\right), & -1.574 < \zeta < 0 \text{ (unstable)} \\ \log\left(\frac{-1.574}{z_{0\mathrm{h}}\zeta}\right) - \Psi_{\mathrm{M1}} + 1.14\left((-\zeta)^{0.3333} - (1.574)^{0.3333}\right), & \zeta < -1.574 \text{ (very unstable)} \\ -5\left(\zeta - z_{0\mathrm{h}}\zeta\right), & 0 < \zeta < 1 \text{ (stable)} \\ \log\left(\frac{\zeta^5}{z_{0\mathrm{h}}\zeta}\right) + 5\left(1 - z_{0\mathrm{h}}\zeta\right) + \zeta - 1, & \zeta > 1 \text{(very stable)} \end{cases} \tag{8}$$

$$
\Psi_{\mathrm{H}}=\begin{cases}2\log\left(\frac{1+x^2}{1+x_0^2}\right), & -1.574<\zeta<0 \text{ (unstable)}\\ \log\left(\frac{-0.465}{z_{\mathrm{Th}}\zeta}\right)-\Psi_{\mathrm{H1}}+0.8\left((-\zeta)^{0.3333}-(0.465)^{0.3333}\right), & \zeta<-1.574 \text{ (very unstable)}\\ -5\left(\zeta-z_{\mathrm{Th}}\zeta\right), & 0<\zeta<1 \text{ (stable)}\\ \log\left(\frac{\zeta^5}{z_{\mathrm{Th}}\zeta}\right)+5\left(1-z_{\mathrm{Th}}\zeta\right)+\zeta-1, & \zeta>1 \text{(very stable)}\end{cases} \tag{9}
$$

where ζ is the stability parameter, $x=(1-16\zeta)^{1/4}$, $x_0=(1-16z_{0\mathrm{h}}\zeta)^{1/4}$, $z_{0\mathrm{h}}=z_0/(z-z_{\mathrm{disp}})$, $z_{\mathrm{Th}}=z_{\mathrm{t0}}/(z-z_{\mathrm{disp}})$, z_0 is the surface roughness and z_{t0} is the roughness for heat, z_{disp} is displacement height, z is the height, Ψ_{M1} and Ψ_{H1} represent the stability function in their first unstable case, respectively.

Supplementary Results

Winter climatology of lead width and length

We have analyzed the size distribution, including lead width, length, and aspect ratio (i.e., length/width), of the wintertime leads over the Pan-Arctic. We used the MODIS derived lead dataset[4], which provides the lead width and length information. The available full data length is from January to April, 2003–2018. The probability distribution functions (PDFs) in Supplementary Fig. 1 suggest that leads range in width from several meters to tens of kilometers and in length from hundreds of meters to hundreds of kilometers. Narrow (2–6 km) and short (20–30 km) leads are the most prevalent, with lead width (length) decreasing with increasing lead width (length). This is consistent with the previous studies[4–7], in which a negative exponential distribution or a power law was used for lead width parameterization.

Winter climatology of lead area fraction and frequency

We have also performed analyses for a ten-year climatology of wintertime lead area fraction, using the full length of the available AMSR-E derived lead dataset (November–April, 2002–2011). Supplementary Fig. 2a shows that the climatological lead area fraction is below 2.5% over the central Arctic, yet exhibits large value (over 30%) in the peripheral seas. For the focused region in our study, i.e., 200 km offshore of Barrow (71–74.6^{o}N, 151–162°W), the averaged lead area fraction is between 2.5% to 15% (Supplementary Fig. 2b), which is higher than the lead fraction in the central Arctic due to the Beaufort Gyre northeast of the Barrow site. We have also examined the climatology of lead occurrence (Supplementary Fig. 3), and found the frequency percentage of lead occurrence is high over the regions where the average lead fraction is large. For our focused domain near Barrow (Supplementary Fig. 3b and d), in terms of lead fraction exceeding 0 (50), most of the area has a lead occurrence frequency greater than 12.5% (2.5%). Leads are much more frequent (over 30%) near the coastal regions. The results are in good agreement with previous studies[6], which estimated that the winter lead fraction of the central Arctic is around 2–3% and 6–9% in the peripheral seas.

Time scales for lead opening and freezing

The time scale upon which a lead opens and subsequently freezes over depends mainly on the lead width and ambient conditions, such as the wind speed, wind direction, and air temperature[8]. Previous modeling studies indicate that for a 500 m-wide lead, it takes around 8 hours to cover the open water under the conditions of 10 m s^{-1} for wind speed and -40°C for air temperature[8,9]. Wider leads take longer time to entirely freeze over and can last up to several days.

Importance of leads in Arctic boundary layer clouds and surface energy budget

Leads or cracks in the sea ice are the main source of moisture for the wintertime boundary layer. Low-level clouds produced by leads are thus an important formation mechanism for boundary layer clouds [10]. Though leads are mostly narrow and maintain for several hours or several days, the existence of leads can affect wintertime low-level clouds amount [11]. Influence from wider leads is even larger. For example, a massive ice crystal plume originating from a lead was observed to reach 4 km in altitude and to extent downwind for about 250 km [12]. Aircraft observations [13] over the Arctic leads also demonstrate the effects of leads on the boundary layer, including air temperature, low level wind speed, humidity, and boundary layer height. Additionally, modeling studies [14] found a 1% decrease in lead fraction may have up to 3.5 °C warming effect on the 10-m air temperature, given that sea ice concentration is greater than 90%. As for the surface energy balance, these lead-produced clouds are especially important in the Arctic longwave budget. Because of the frequent temperature inversions in the winter Arctic, clouds emit at temperatures that are warmer than the underlying ice surface. Heat exchange at the open water surface of a lead is 2 orders of magnitude greater than that through surrounding snow-covered pack ice, so even a small percentage of these features and of thin ice can dominate regional heat budgets in winter [15,16].

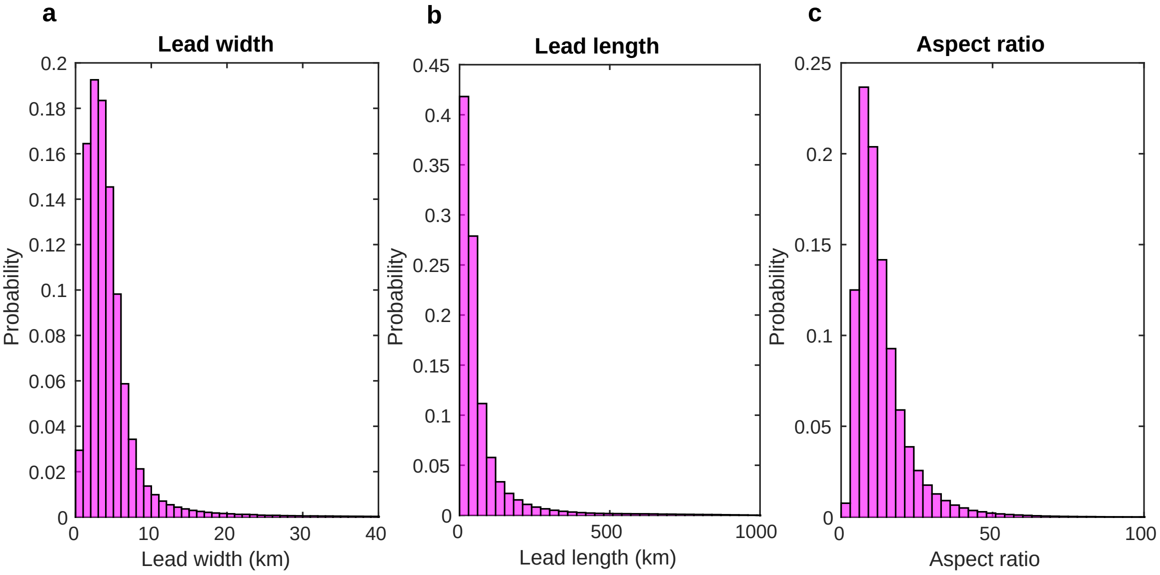

Supplementary Fig. 1 | MODIS derived lead width, length and aspect ratio. a, The probability distribution function (PDF) of the lead width occurred over the Pan-Arctic during January to April, 2003–2018. **b-c,** The same as **a** but for lead length and aspect ratio, respectively.

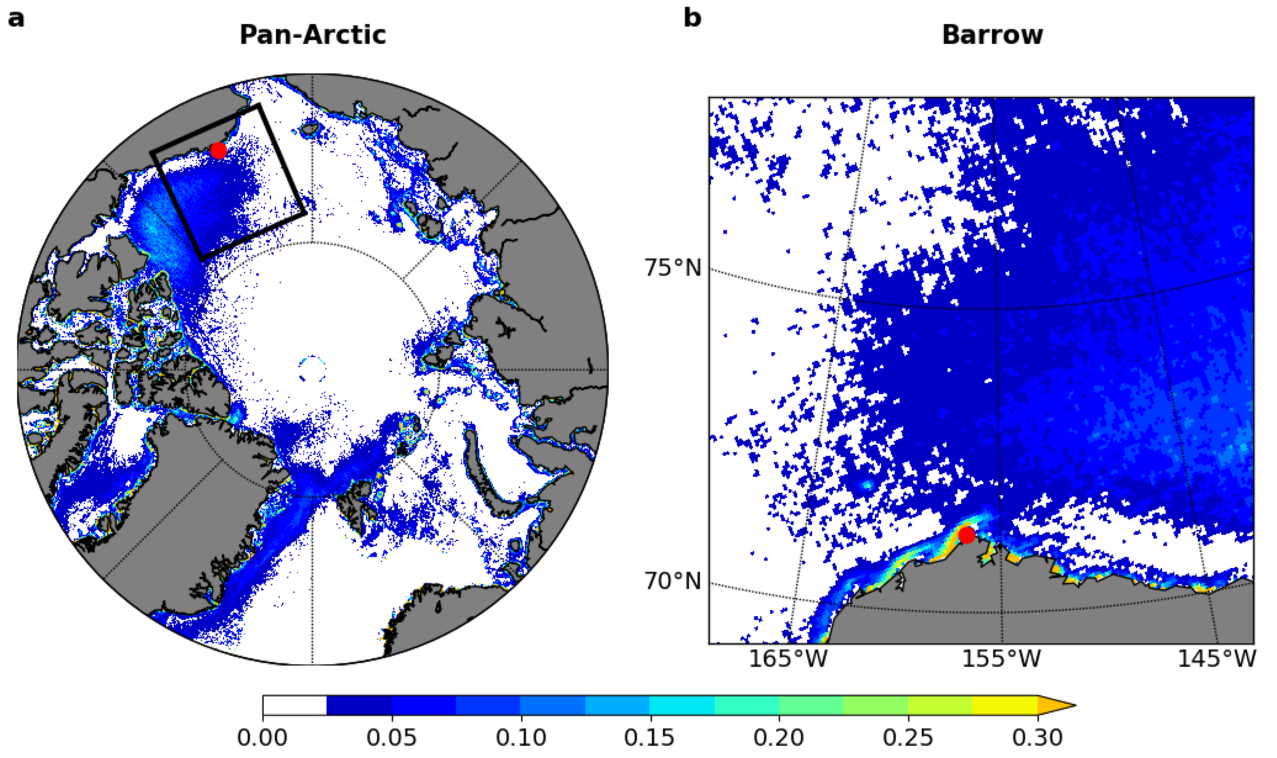

Supplementary Fig. 2 | AMSR-E derived lead area fraction. a, Average lead area fraction over Pan-Arctic during November to April, 2002–2011. **b,** The same as **a** but for a regional region offshore of Barrow, as indicated by the black box in **a**.

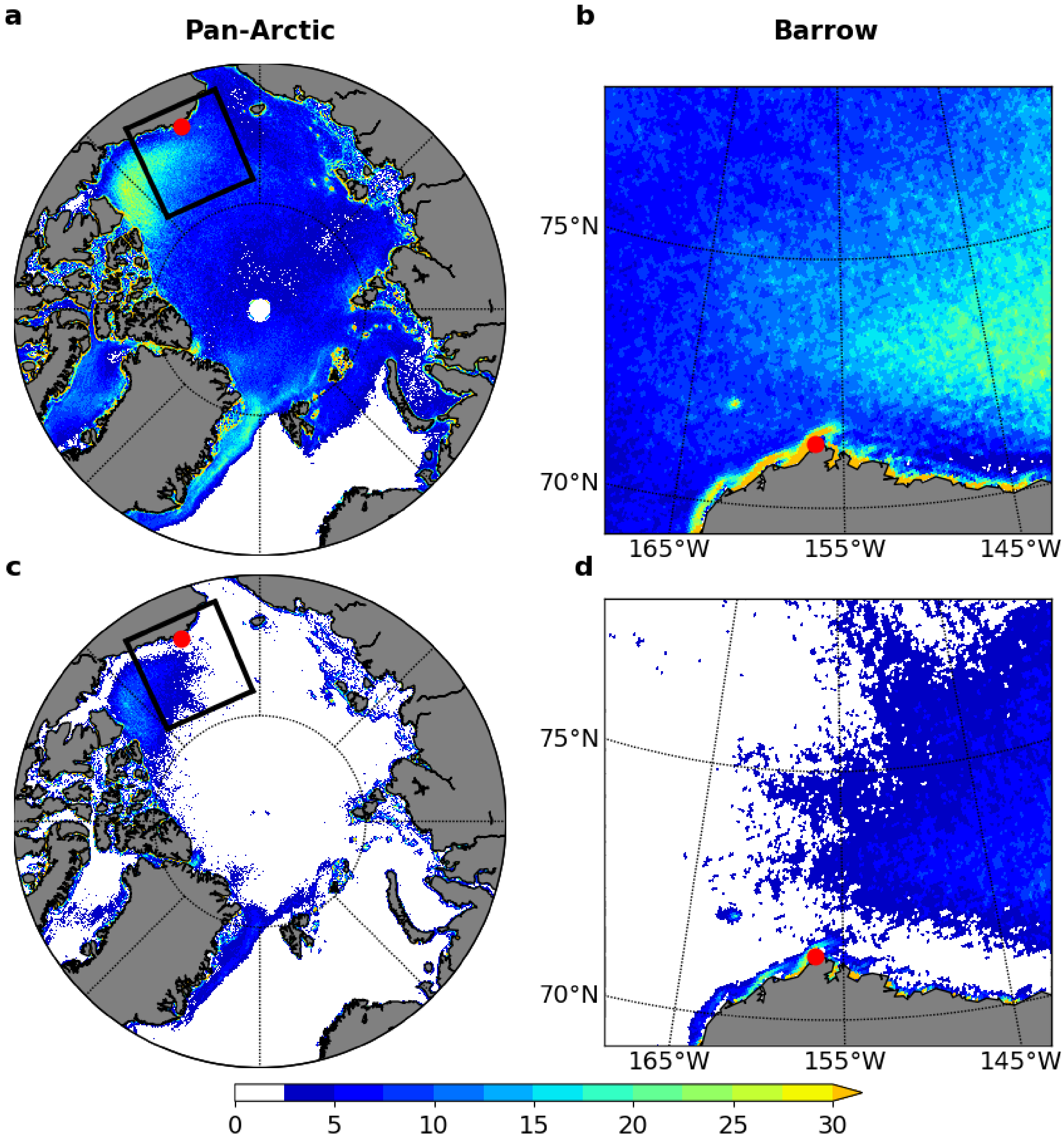

Supplementary Fig. 3 | AMSR-E derived lead frequency. a, Frequency percentage (%) of lead occurrence (with lead area fraction greater than 0) over the Pan-Arctic during November to April, 2002–2011. **b,** The same as **a** but for a regional region offshore of Barrow, as indicated by the black box in **a**. **c-d,** The same as **a-b** but for lead occurrence frequency with lead area fraction greater than 0.5.

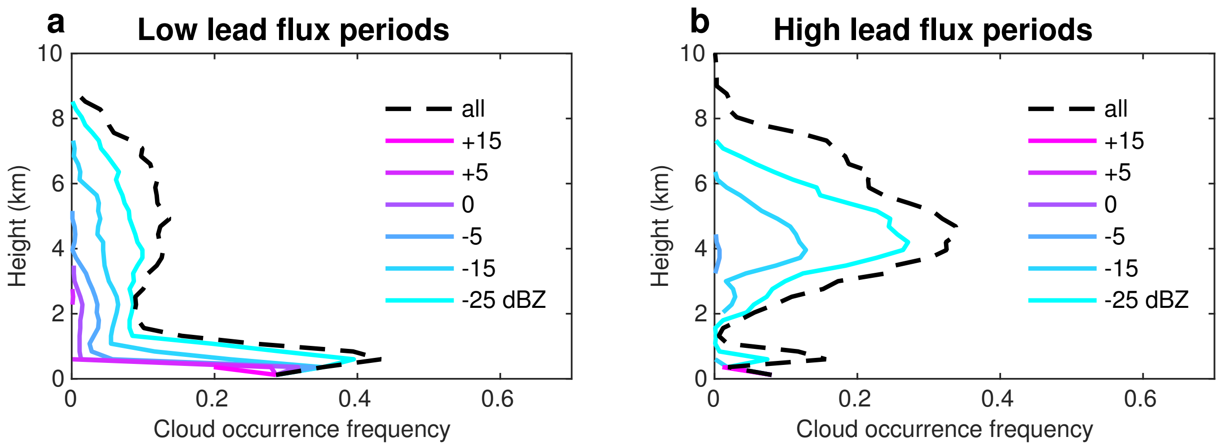

Supplementary Fig. 4 | CloudSat-CALIPSO derived cloud occurrence frequency. **a,** The vertical frequency distribution of cloud occurrence as a function of minimum reflectivity (dBZ) for the low lead flux periods. **b,** The same as **a** but for the high lead flux periods.

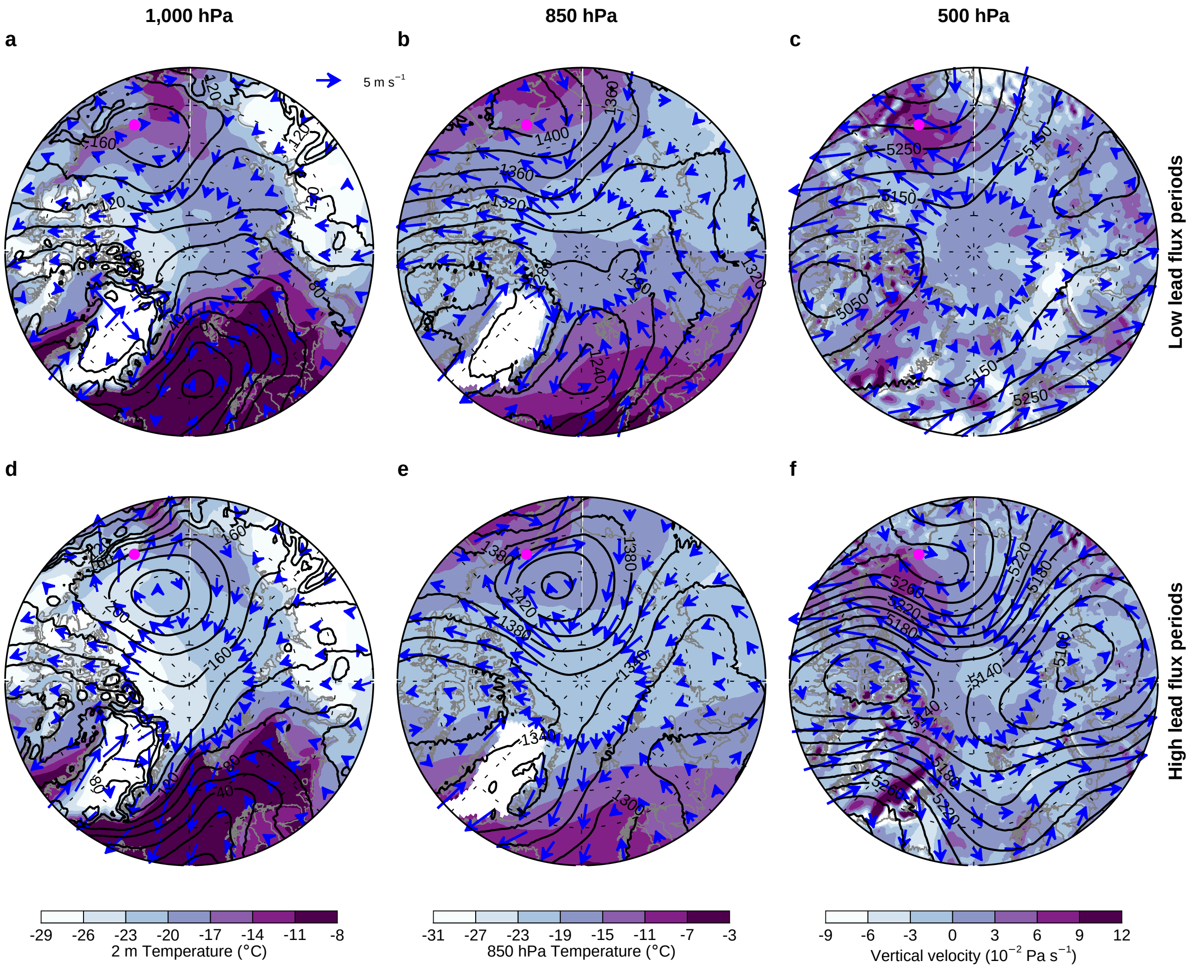

Supplementary Fig. 5 | Large-scale composite synoptic conditions for the pan-Arctic. a-f, The same as Fig. 2 but for the pan-Arctic (poleward of 65°N).

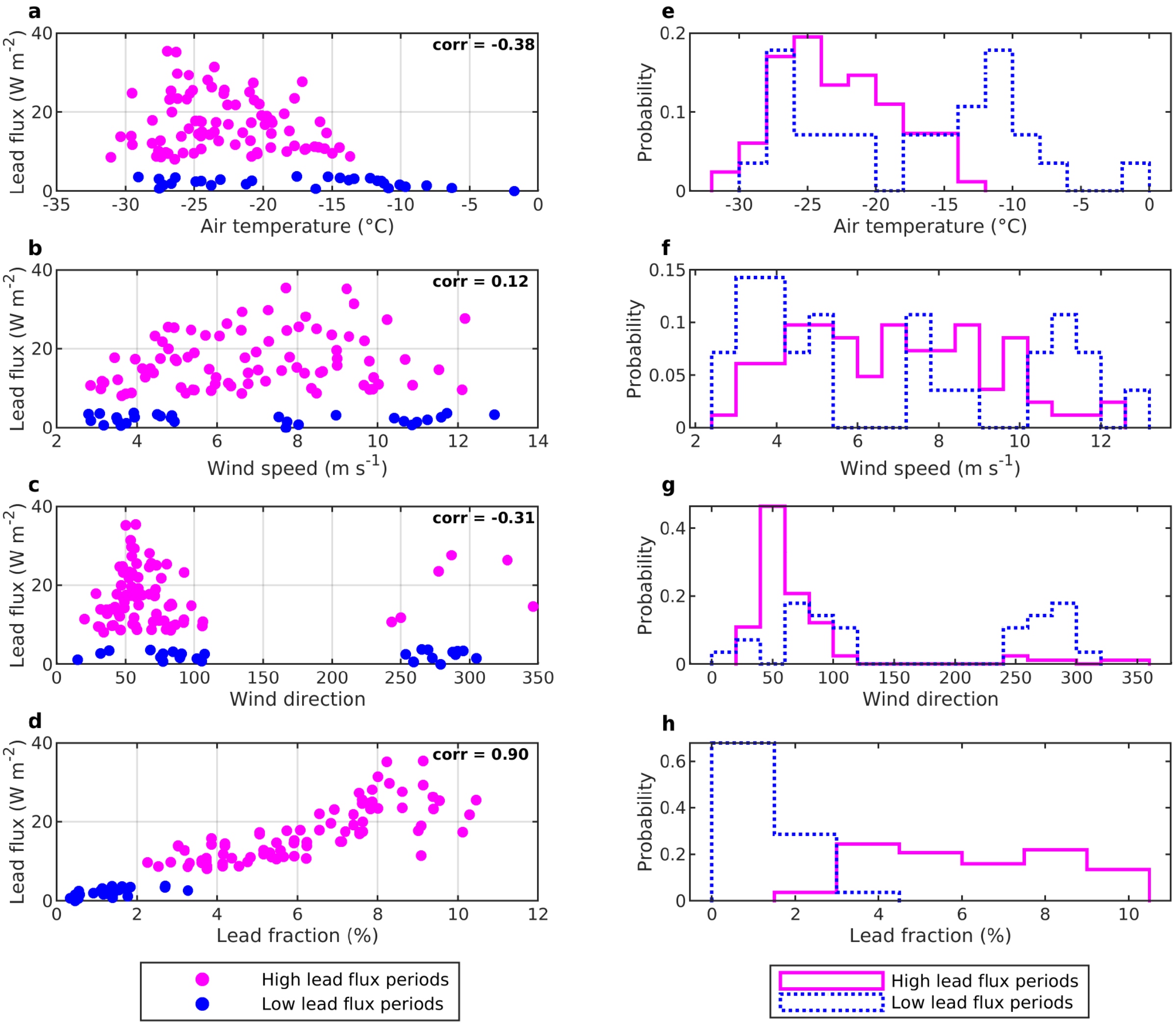

Supplementary Fig. 6 | Distribution of synoptic conditions at Barrow and lead area fraction. a-d, Scatterplots of 2-m air temperature, wind speed, wind direction, and lead fraction versus large-scale lead flux, respectively. Magenta (blue) represents high (low) lead flux periods. **e-h,** The corresponding probability distribution function for the 2-m air temperature, wind speed, wind direction, and lead fraction.

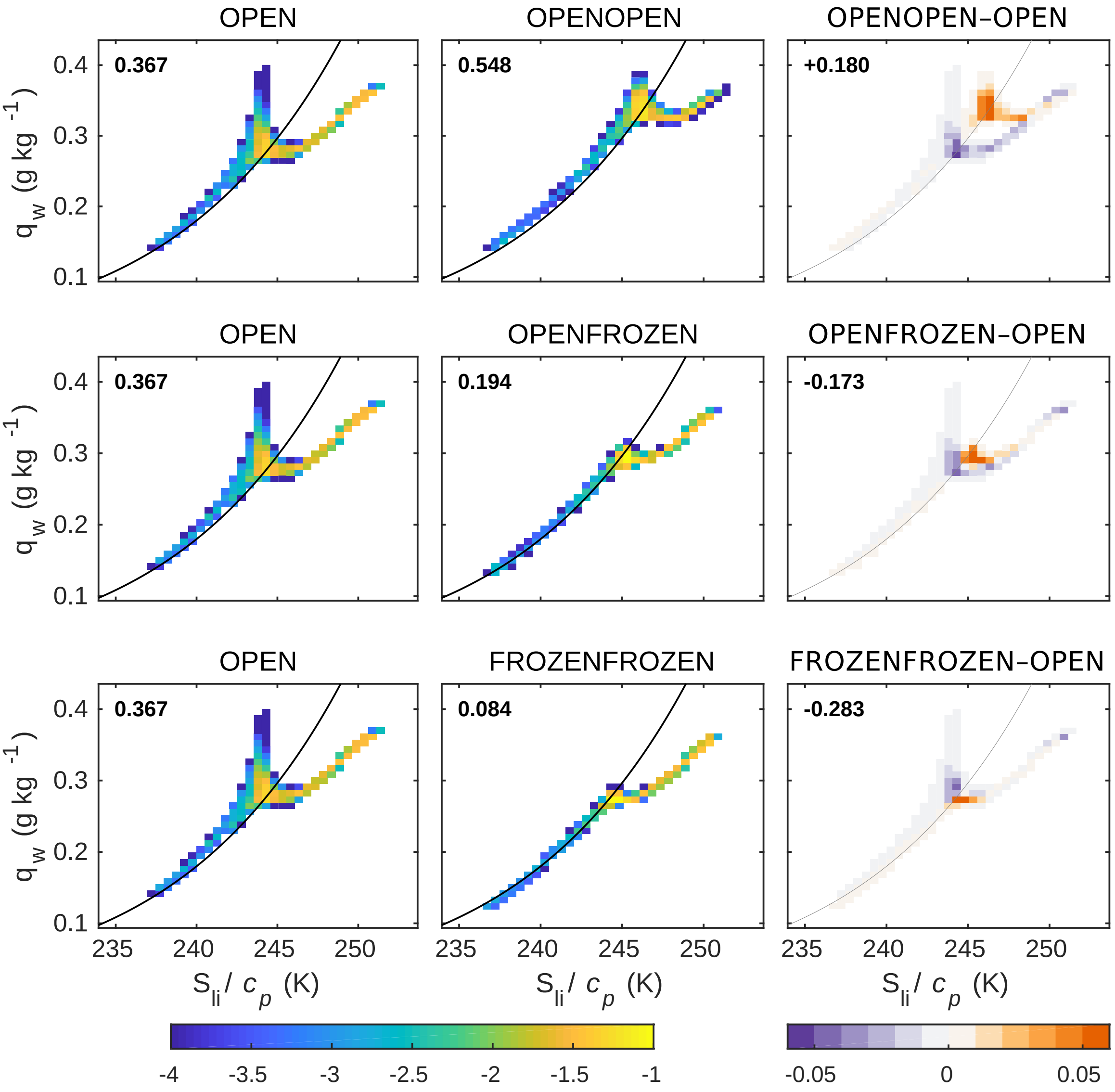

Supplementary Fig. 7 | Mixing diagram of the four cases and the difference between high and low lead fraction cases. The same as Fig. 6 but shows the 2D PDFs of thermodynamic state characterized by liquid-ice static energy (S_{li}/c_p) and total water mixing ratio (q_w) for all four cases as well as the differences between OPEN and the other three high lead fraction cases. Results in the first two columns are shown in log10 scale. The numbers on the top left are the fractions of cloudy volume in each case and the differences between the OPEN case and the other three cases.

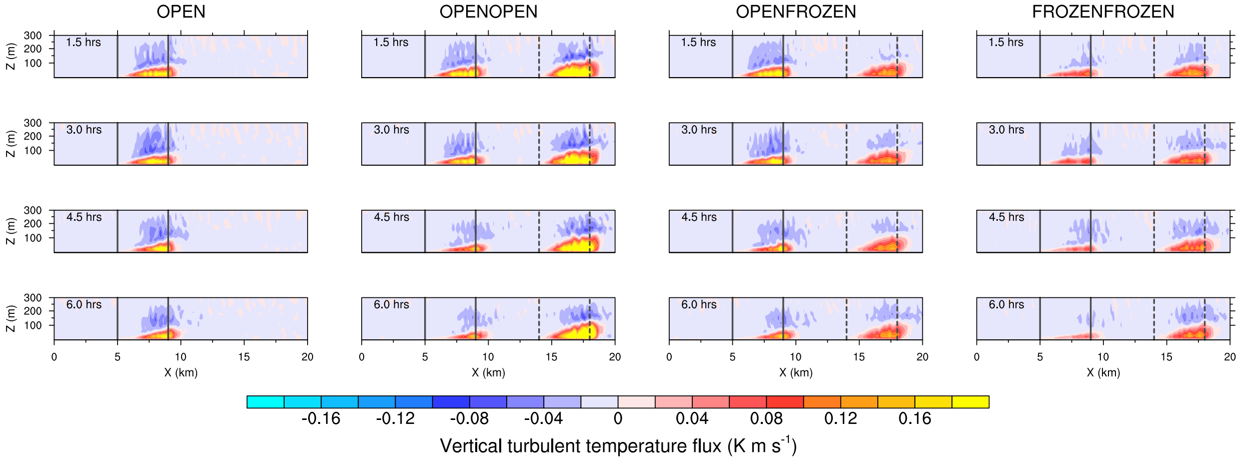

Supplementary Fig. 8 | x-z profiles of the vertical turbulent temperature flux. Vertical turbulent temperature flux from the OPEN, OPENOPEN, OPENFROZEN, and FROZENFROZEN cases at simulation hours of 1.5, 3.0, 4.5, and 6.0, respectively. All results are averaged along the y direction. The upstream open lead extends from $x = 5$ km to $x = 9$ km, as indicated by the two vertical solid lines in the domain, and the downstream open or frozen lead extends from $x = 14$ km to $x = 19$ km, as indicated by the two vertical dashed lines.

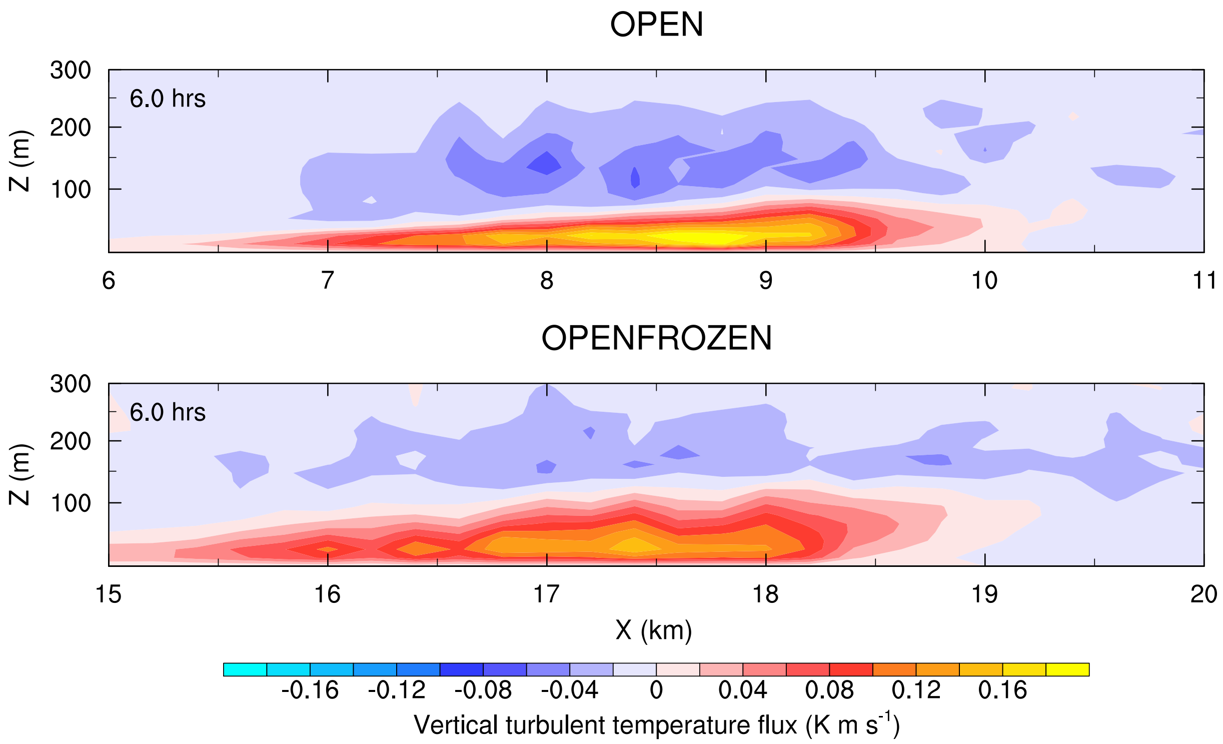

Supplementary Fig. 9 | Zoomed x-z profiles of the vertical turbulent temperature flux. Vertical turbulent temperature flux of a zoomed portion from OPEN and OPENFROZEN case at simulation hours of 6. x focuses near the open lead area (6 to 11 km) in the OPEN case, and near the downstream frozen lead (15 to 20 km) in the OPENFROZEN case.

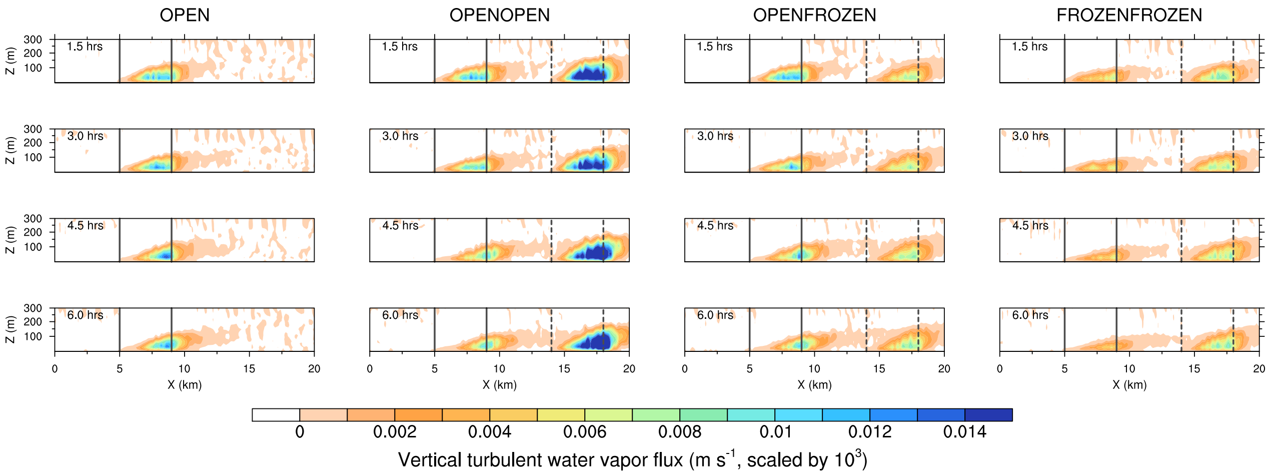

Supplementary Fig. 10 | x-z profiles of the vertical turbulent water vapor flux. The same as Supplementary Fig. 8, but for the vertical turbulent water vapor flux.

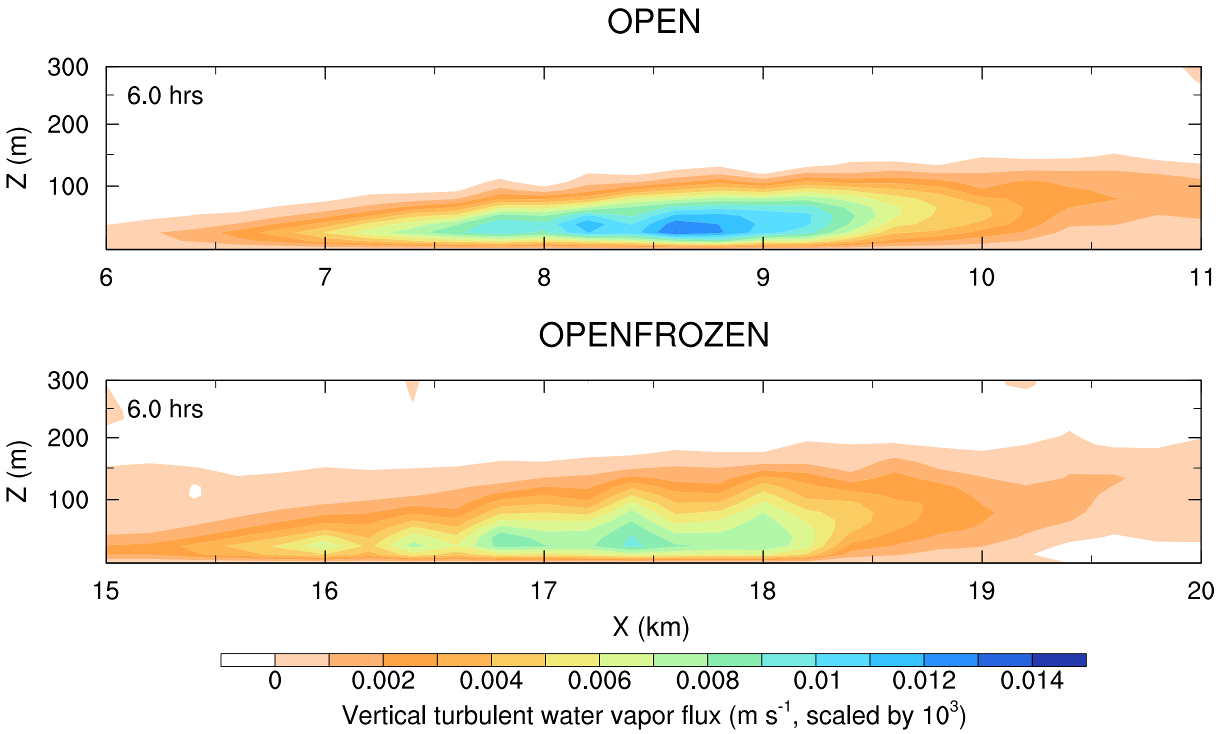

Supplementary Fig. 11 | Zoomed x-z profiles of the vertical turbulent water vapor flux. The same as Supplementary Fig. 9, but for the vertical turbulent water vapor flux.

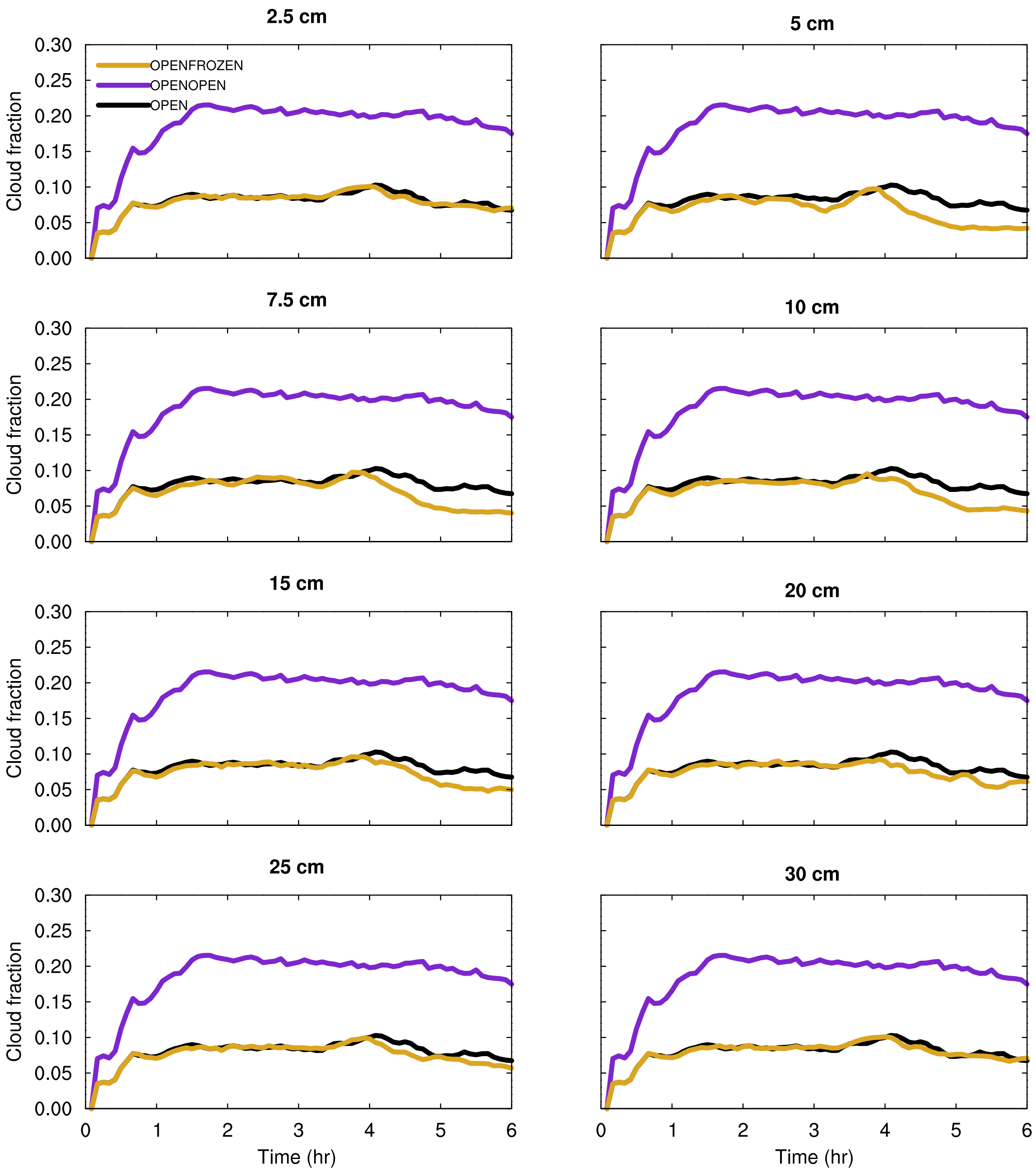

Supplementary Fig. 12 | Time series of cloud coverage with different frozen ice thickness. The same as Fig. 4 but for OPENFROZEN case with eight different frozen ice thickness, ranging from 2.5 cm up to 30 cm. The 5 cm case is the same as Fig. 4.

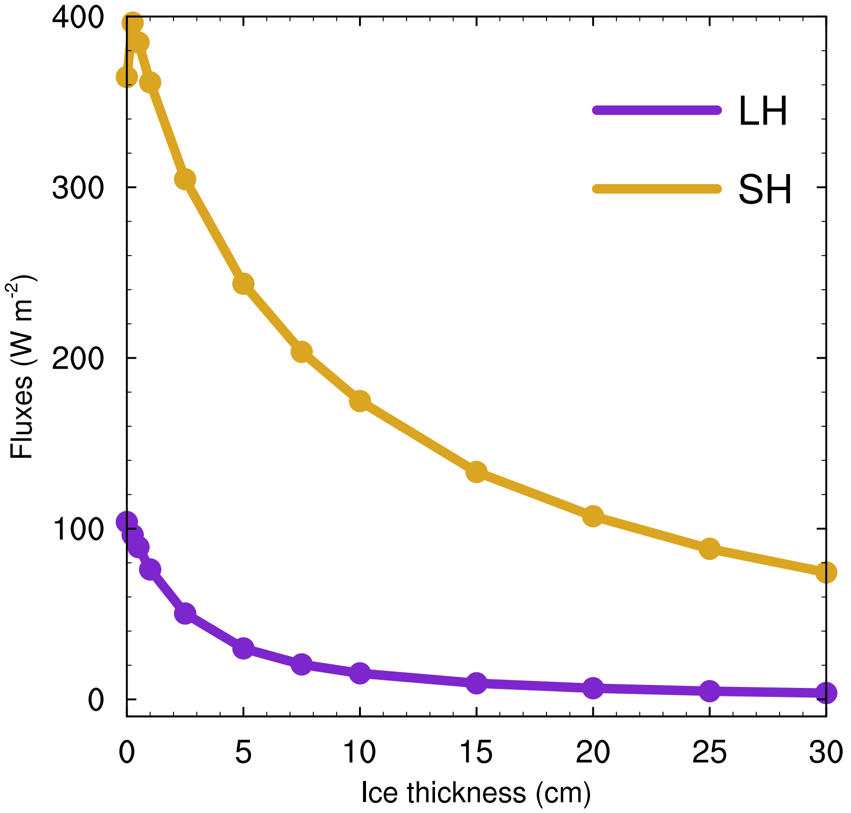

Supplementary Fig. 13 | Surface turbulent fluxes as a function of ice thickness. Surface latent heat flux (LH) and sensible heat flux (SH) averaged over the frozen lead and as a function of frozen ice thickness. Results are from the OPENFROZEN case with eight different frozen ice thickness, corresponding to the cloud coverage shown in Supplementary Fig. 12.

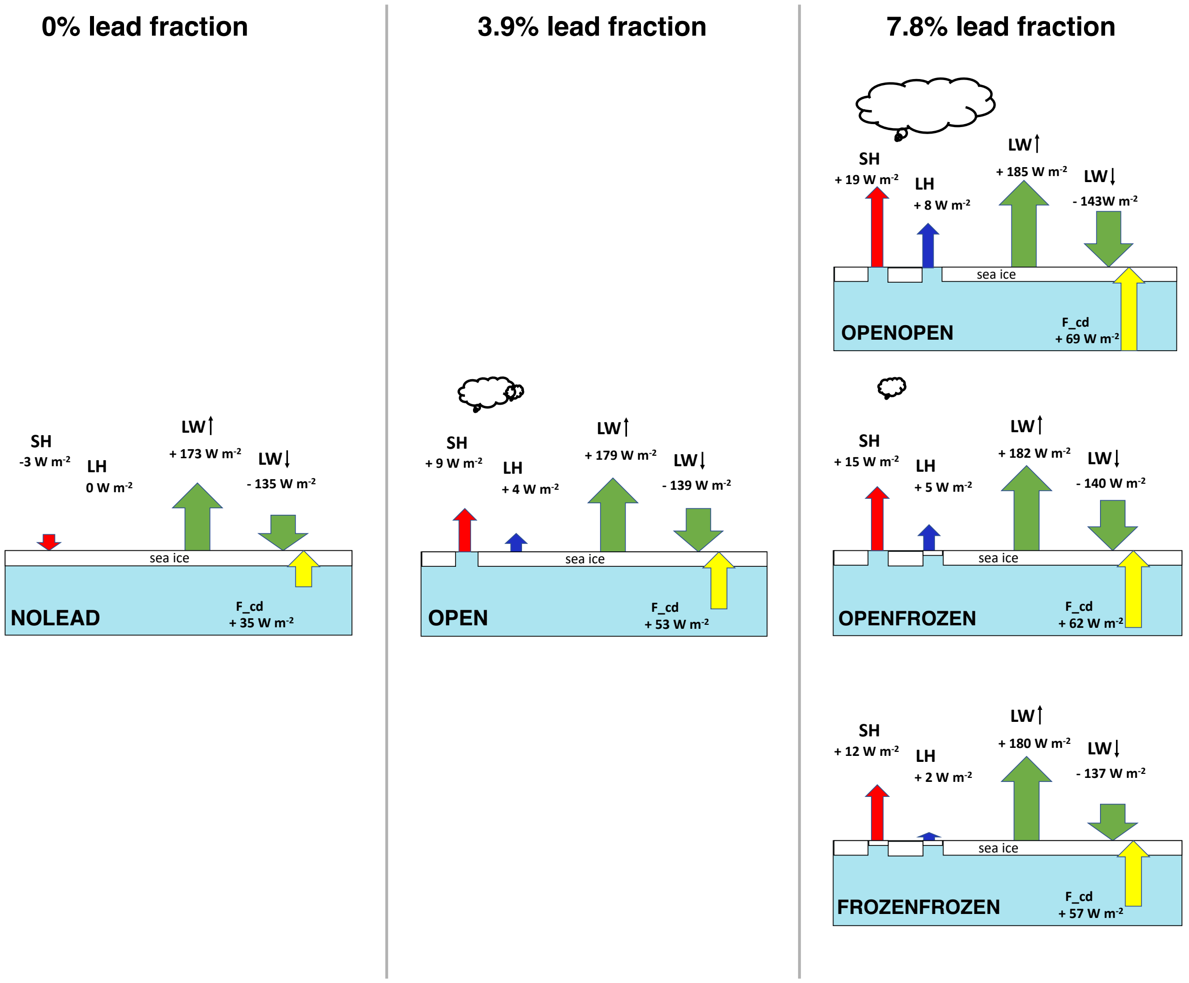

Supplementary Fig. 14 | Cartoon schematic for the large-scale surface energy budget in each case. The entire domain averaged surface sensible heat flux (SH), latent heat flux (LH), upwelling longwave radiative flux (LW↑), downwelling longwave radiative flux (LW↓), and the conductive heat flux from the bottom ocean (F$_cd$). All the cases, including the NOLEAD, are categorized by the lead fraction. Upward flux is defined as positive.

Supplementary Table 1 | Dataset used in the Methods

Dataset	Source	Variables
Radiosonde at Barrow	ARM SONDE[1]	12-hourly relative humidity at 10-m intervals
Surface measurements at Barrow	NOAA[2]/ESRL[3]/GMD[4]/BRW[5]	Hourly temperature, pressure, relatively humidity, wind speed and wind direction
AMSR-E derived lead area fraction	ICDC[6]<br>University of Hamburg	Daily lead area fraction
Ground-based cloud radar data at Barrow	ARM MMCR	Radar reflectivity
CloudSat CPR radar and CALIPSO lidar data	CloudSat DPC[7]	Radar reflectivity,<br>cloud fraction
Atmospheric reanalysis from MERRA-2	GES[8] DISC[9]	Hourly wind speed, wind direction, temperature, geopotential height
NCEP/NCAR Reanalysis 1	NOAA/ESRL/PSD[10]	6-hourly geopotential height
MODIS derived lead data	SSEC[11]<br>University of Wisconsin-Madison	Daily lead width and length

[1] Balloon-Borne Sounding System, [2] National Oceanic and Atmospheric Adminstration, [3] Earth System Research Laboratory, [4] Global Monitoring Division, [5] Barrow Atmospheric Baseline Observatory, [6] Integrated Climate Data Center, [7] Data Processing Center, [8] Goddard Earth Sciences, [9] Data and Information Services Center, [10] Physical Sciences Division, [11] Space Science and Engineering Center

Supplementary References

1. Andreas, E. L. & Cash, B. A. Convective heat transfer over wintertime leads and polynyas. *J. Geophys. Res. Oceans* **104**, 25721–25734 (1999).

2. Marcq, S. & Weiss, J. Influence of sea ice lead-width distribution on turbulent heat transfer between the ocean and the atmosphere. *The Cryosphere* **6**, 143–156 (2012).

3. Andreas, E. L. & Murphy, B. Bulk transfer coefficients for heat and momentum over leads and polynyas. *J. Phys. Oceanogr.* **16**, 1875–1883 (1986).

4. Hoffman, J. P., Ackerman, S. A., Liu, Y. & Key, J. R. The detection and characterization of Arctic sea ice leads with satellite imagers. *Remote Sensing* **11**, 521 (2019).

5. Key, J. & Peckham, S. Probable errors in width distributions of sea ice leads measured along a transect. *J. Geophys. Res. Oceans* **96**, 18417–18423 (1991).

6. Lindsay, R. W. & Rothrock, D. A. Arctic sea-ice leads from advanced very high-resolution radiometer images. *J. Geophys. Res. Oceans* **100**, 4533–4544 (1995).

7. Tschudi, M. A., Curry, J. A. & Maslanik, J. A. Characterization of springtime leads in the Beaufort/Chukchi Seas from airborne and satellite observations during FIRE/SHEBA. *J. Geophys. Res. Oceans* **107**, 8034 (2002).

8. Bauer, J. & Martin, S. A model of grease ice growth in small leads. *J. Geophys. Res. Oceans* **88**, 2917–2925 (1983).

9. Pinto, J. O., Alam, A., Maslanik, J. A., Curry, J. A. & Stone, R. S. Surface characteristics and atmospheric footprint of springtime Arctic leads at SHEBA. *J. Geophys. Res. Oceans* **108**, 27 (2003).

10. Curry, J. A., Meyer, F. G., Radke, L. F., Brock, C. A. & Ebert, E. Occurrence and characteristics of lower tropospheric ice crystals in the Arctic. *Int. J. Climatol.* **10**, 749–764 (1990).

11. Pinto, J. O., Curry, J. A. & Mcinnes, K. L. Atmospheric convective plumes emanating from leads 1. thermodynamic structure. *J. Geophys. Res. Oceans* **100**, 4621–4631 (1995a).

12. Schnell, R. C. *et al.* Lidar detection of leads in Arctic sea ice. *Nature* **339**, 530–532 (1989).

13. Tetzlaff, A., Lüpkes, C. & Hartmann, J. Aircraft-based observations of atmospheric boundary-layer modification over Arctic leads. *Q. J. R. Meteorol. Soc.* **141**, 2839–2856 (2015).

14. Lüpkes, C., Vihma, T., Birnbaum, G. & Wacker, U. Influence of leads in sea ice on the temperature of the atmospheric boundary layer during polar night. *Geophys. Res. Lett.* **35**, L03805 (2008b).

15. Maykut, G. A. Energy exchange over young sea ice in the central Arctic. *J. Geophys. Res.* **83**, 3646–368 (1978).

16. Ledley, T. S. For a lead-temperature feedback in climatic variation. *Geophys. Res. Lett.* **15**, 36–39 (1988).
